# Coronary artery-pulmonary artery fistula: case report

**DOI:** 10.1186/1476-7120-5-19

**Published:** 2007-04-11

**Authors:** Ilaria Quatrini, Valerio Zacà, Sergio Mondillo

**Affiliations:** 1 Department of Cardiology, University of Siena, Siena, Italy

## Abstract

**Background:**

Coronary artery fistulas are rare congenital or acquired coronary artery anomalies that can originate from any of the three major coronary arteries and drain in all the cardiac chambers and great vessels.

**Case presentation:**

An 11-year-old boy was referred for evaluation of an exertional dyspnoea. He reported recent history of few episodes of shortness of breath associated with moderate entity physical activity. At physical examination a mild continuous murmur could be heard mainly at the level of the second intercostal space of the left parasternal area. A transthoracic echocardiogram showed a continuous flow at color Doppler analysis in the high parasternal short axis view, originating from a small entry site on the wall of the main pulmonary artery. A selective left coronary angiography revealed a fistula connecting the proximal portion of the left anterior descending coronary artery with the main pulmonary artery.

**Conclusion:**

A combination like the one described in the present case is unusual since fistulas originate from the left coronary artery in about 35% of cases and drainage into the pulmonary artery occurs in only 17%.

## Background

Coronary artery fistulas are rare congenital or acquired coronary artery anomalies that can originate from any of the three major coronary arteries and drain in all the cardiac chambers and great vessels [1,2].

## Case presentation

**Figure 1:**
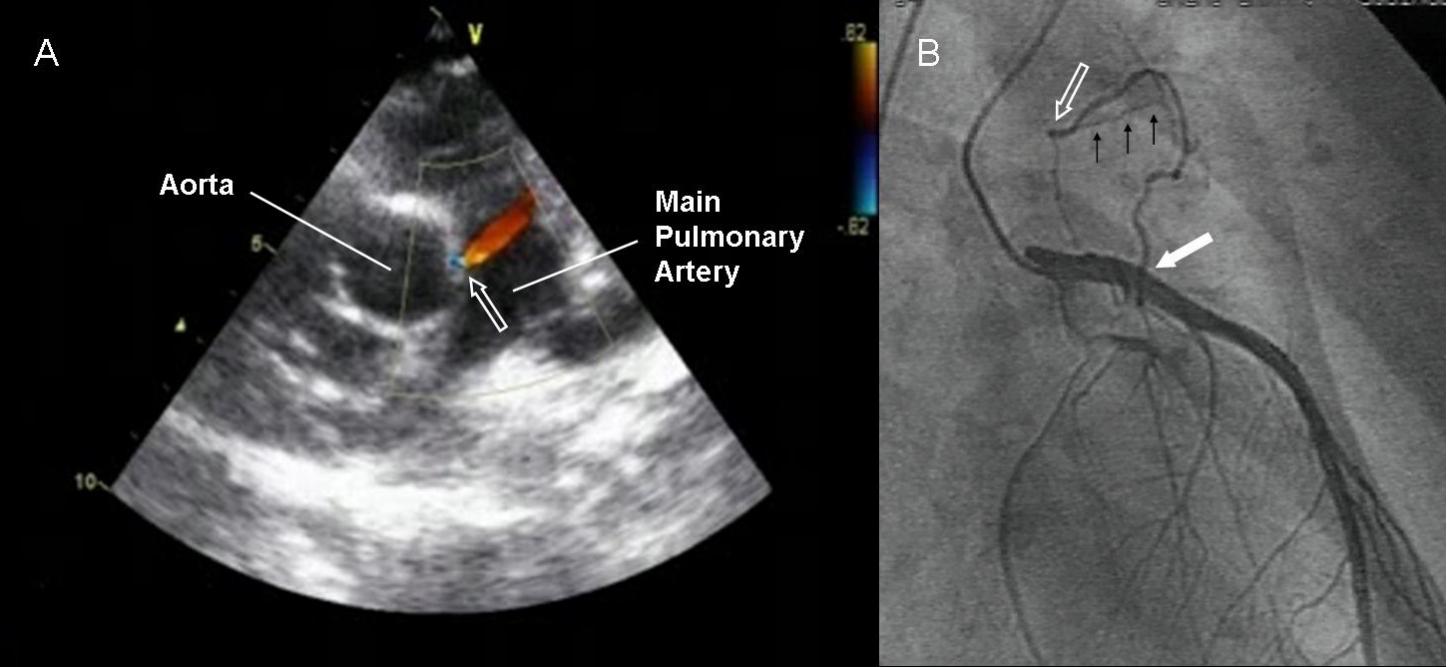


An 11-year-old boy was referred for evaluation of an exertional dyspnoea. He reported recent history of few episodes of shortness of breath associated with moderate entity physical activity. At physical examination a mild continuous murmur could be heard mainly at the level of the second intercostal space of the left parasternal area. A transthoracic echocardiogram (Figure 1A) showed a continuous flow at color Doppler analysis in the high parasternal short axis view, originating from a small entry site on the wall of the main pulmonary artery (arrow). A selective left coronary angiography (Figure 1B) revealed a fistula connecting the proximal portion of the left anterior descending coronary artery (solid white arrow) with the main pulmonary artery (blank white arrow); black arrows indicated drainage into the pulmonary circulation.

## Conclusion

Most fistulas originate from the right coronary artery or the left anterior descending and commonly drain in low-pressures structures including right-sided chambers, pulmonary artery, superior cava vein and coronary sinus [1,2]. A combination like the one described in the present case is unusual since fistulas originate from the left coronary artery in about 35% of cases and drainage into the pulmonary artery occurs in only 17% [1,2].

## Competing interests

The author(s) declare that they have no competing interests.

## Authors' contributions

IQ collected the data relative to the Case Report; VZ composed the draft of the manuscript; SM is head of the Echo Lab and conceived the Case report and participated in the coordination, data analysis and elaboration and drafting of the manuscript. All authors read and approved the final manuscript.
